# The Prevalence and Impact of Allergic Rhinitis on Asthma Exacerbations in Asthmatic Adult Patients in the Qassim Region of Saudi Arabia: A Cross-Sectional Study

**DOI:** 10.7759/cureus.44997

**Published:** 2023-09-10

**Authors:** Sami M Alrasheedi, Khalid M Alkhalifah, Shoug Alnasyan, Raghad R Alwattban, Rahaf A Alsubhi, Rana I Alsamani, Yasmeen A Alfouzan

**Affiliations:** 1 Medicine, Unaizah College of Medicine and Medical Sciences, Qassim University, Unaizah, SAU; 2 College of Medicine, Qassim University, Qassim, SAU

**Keywords:** ar, impact, prevalence, asthma, allergic rhinitis

## Abstract

Objective

We aim to evaluate the prevalence and impact of allergic rhinitis comorbidity in asthmatic patients in the Qassim region and identify whether rhinitis affects asthma control.

Methods

This is an observational cross-sectional study on asthmatic adults who live in the Qassim region of Saudi Arabia. An online questionnaire was distributed through social media. The questionnaire is composed of the validated Arabic versions of the Score for Allergic Rhinitis (SFAR) questionnaire, the Allergic Rhinitis and Its Impact on Asthma (ARIA) guidelines, and the Asthma Control Test (ACT) questionnaire.

Results

The total number of participants was 380; however, after 98 were excluded, 282 asthmatic patients were included in this study. Of them, 33% had allergic rhinitis. Females constitute 67% of the study participants, while males comprise 33%. The findings of the study indicate that there is a significant association between allergic rhinitis and asthma control in the Qassim region. Symptoms such as runny nose, sneezing, and nasal obstruction are significantly associated with poor asthma control (p = 0.006). Having a known family history of asthma, eczema, or allergic rhinitis is significantly associated with worse asthma control (0.004).

Conclusion

In summary, this study found a high prevalence of rhinitis symptoms comorbidity in asthmatic patients in the Qassim region. Overall, the study established the existence of a relationship between allergic rhinitis and asthmatic control. Symptoms such as a runny nose, sneezing, and nasal obstruction are significantly associated with allergic rhinitis and asthmatic symptoms. However, there is no significant association between nose problems that occur in specific seasons or months and allergic rhinitis and asthmatic symptoms, suggesting that seasonality may not have a strong impact on asthma control. House dust mite allergies have a borderline significant association with allergic rhinitis and asthmatic symptoms. Having a family history of asthma, eczema, or allergic rhinitis is associated with allergic rhinitis and asthmatic symptoms, thereby indicating a significant impact on asthma control.

## Introduction

Asthma is known as a chronic respiratory disease, and it is a widespread disease in the world. The World Health Organization defines it as having frequent attacks of wheezing, shortness of breath, and coughing. However, the seriousness of asthma varies from person to person [1]. Allergic rhinitis (AR), defined as an inflammation of the nasal mucosa caused by exposure to allergens like pollen, trees, weeds, and house dust mites, is also known as a chronic respiratory disease [2]. Rhinorrhea, nasal itching, nasal congestion, and sneezing are known characteristics of AR [3]. Moreover, it has been known that asthma and AR are related. The pathophysiology of both diseases is largely influenced by the immune system. Recent research found that asthma severity could increase the likelihood of AR by triggering an inflammatory response in the upper respiratory tract [4].

According to some studies, the severity of AR and asthma may be related, with the frequency and severity of AR increasing with the severity of asthma [5,6]. In the Riyadh region, a cross-sectional study was conducted which suggests a positive association between asthma and AR control [7]. When asthma and AR coexist, this condition may require more hospitalizations and doctor visits for asthma-related issues, incurring higher costs overall [8-10].

This cross-sectional research aims to evaluate the prevalence and severity of rhinitis comorbidity in asthmatics patients utilizing asthma medication in the Qassim region and identify whether rhinitis affects control.

## Materials and methods

Study design

This is a questionnaire-based observational cross-sectional study.

Study setting

This comprises of an online questionnaire for asthmatic adults living in the Qassim region of Saudi Arabia.

Sample size

The sample size was calculated by the Raosoft calculator (Raosoft Inc., Seattle, Washington) based on the population size in the Qassim region (approximately 38,907) [11]. With a 95% confidence interval and a 5% margin of error, we had to collect at least 381 participants for this study. In case of any possible data loss, we add 5% as attrition rate, and the total sample size required is 400 participants.

Sampling technique

Participants were recruited by non-probability convenience sampling method.

Inclusion and exclusion criteria

Inclusion criteria

Participants who have Saudi nationality, are Qassim region residents, are aged 14-70 years, and have been diagnosed clinically with asthma for more than six months were included.

Exclusion criteria

Participants with chronic obstructive pulmonary disease (COPD), pulmonary fibrosis, bronchitis, pneumonia, or who had been diagnosed with COVID-19 in the last six months were excluded.

Data collection methods

After gaining ethical approval, an online questionnaire was distributed among the chosen population to be filled out voluntarily through social media. Patients were asked whether they agreed to participate before filling out the questionnaire. After obtaining permission, we used a questionnaire from a previous study which was taken completely in both languages (Arabic and English) [7]. It starts first with questions that include and exclude the participants' asthma diagnosis and comorbidity and is composed of the validated Arabic versions of the Score for Allergic Rhinitis (SFAR) questionnaire [12], Allergic Rhinitis and Its Impact on Asthma (ARIA) Guidelines [7], and the Asthma Control Test (ACT) questionnaire [13].

Data management and analysis plan

The pre-analysis data obtained from the questionnaires were organized in a Microsoft Excel file. The Excel file containing the participants’ data was then exported into SPSS Statistics for statistical analysis. Any missing entries in the record were marked as "not replied." If double entries were found, the initial answer was selected to represent the correct value. The chi-square test was used to determine the significant association between categorical variables. The p-value < 0.05 was taken as the fixed point for statistical significance.

Ethical considerations

Qassim University Research Ethics Committee approval was obtained (approval number: 23-41-07) for this study before proceeding with it. All of the information was treated confidentially. The participants signed an electronic informed consent form at the beginning of the questionnaire that explained the purpose of this study. All participant data were kept in coded forms and destroyed once the data collection was complete.

## Results

**Figure 1:**
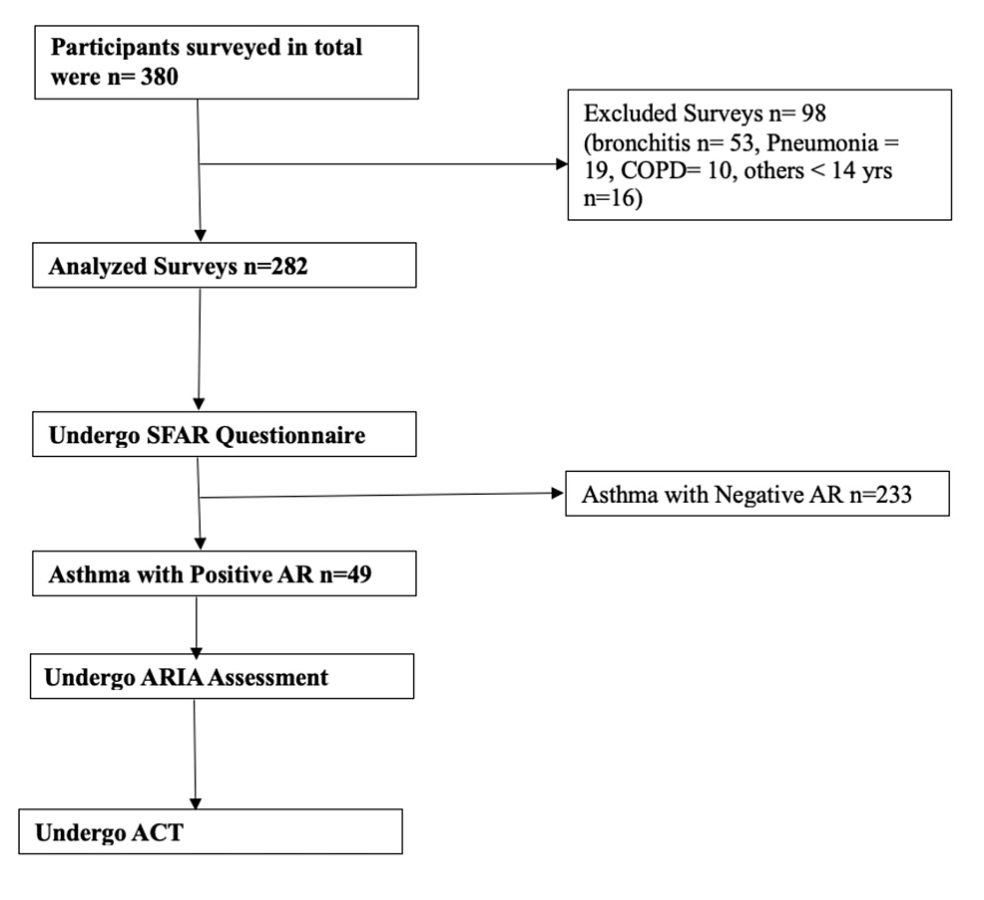
Flow diagram of the study participants AR: Allergic rhinitis; ACT: Asthma Control Test; ARIA: Allergic Rhinitis and Its Impact on Asthma; SFAR: Short-form allergic rhinitis.

Figure 1 shows the flow of participants throughout the study. A total of 380 respondents were viable to carry out the study. However, 98 participants did not meet the threshold to carry out the analysis. Participants with bronchitis (n = 53), pneumonia (n = 19), and COPD (n = 10) were excluded from the study. Others include those who are less than 14 years old (n = 16). Therefore, a total of 282 questionnaires were analyzed representing 74.2% of the participants. The response rate was excellent for carrying out analysis and making conclusions about the study.

The respondents' characteristics are summarized in Table 1.

**Table 1:** Demographic information and clinical features of the participants GERD: Gastroesophageal reflux disease.

Category and features	Frequency and proportion of the respondents, n (%)
Gender	
Male	93 (33%)
Female	189 (67%)
BMI	
Underweight (Below 18.5)	18 (6.38%)
Normal weight (18.5–24.9)	144 (51.06%)
Overweight (25.0–29.9)	79 (28.01%)
Obesity (30.0 and above)	41 (14.53)
Chronic disease suffering	
I don’t have any chronic disease	185 (65.6)
GERD or gastric reflux	15 (5.3%)
Diabetes	10 (3.5%)
Thyroid disease and heart disease	10 (3.5%)
Blood pressure and high blood fats	10 (3.5%)
Others	42 (14.5%)
Age	
14-29 years	189 (67%)
30-45 years	56 (19.9%)
41-56 years	33 (11.7%)
57-70 years	4 (1.4%)
Smoking status	
Ex-smoker	22 (7.8%)
Current smoker	15 (5.3%)
Never smoked	245 (86.9%)
Education	
Primary school and below intermediate school	4 (1.4%)
High school	74 (26.2%)
Middle school	8 (2.8%)
College and above	196 (69.5%)
Diagnosed with allergic rhinitis by a physician	
Yes	93 (33%)
No	189 (67%)
Treatment medication used	
Intranasal corticosteroids	47 (16.7%)
Antihistamine (oral or intranasal)	41 (14.5%)
Leukotriene receptor antagonist (LTRA)	3 (1.1%)
I am not taking any medication	170 (60.3%)

Association between SFAR and asthmatic control

Table 2 shows the association between SFAR and asthmatic symptom control. All those diagnosed with asthma (n = 282) are subjected to SFAR assessment in order to determine some common symptoms for AR and asthmatic patients and to get the respondents who tested positive for allergy (Skin Prick Test/SPT, IgE) so that they can be used to test the association between AR severity and asthmatic control. The results from the chi-square test show that there is a statistically significant association between nasal symptoms, runny nose, sneezing, and nasal obstruction (p = 0.006) among AR and asthmatic patients. On the other hand, there is no statistically significant association between nose problems related to certain seasons or months among AR and asthmatic patients (p = 0.264). This suggests that the seasonality of AR may not have a strong impact on asthma control. There is no statistically significant association between the triggering factors (p = 0.085). A statistically significant association is found between having a family member with asthma, eczema, or AR and having a similar scenario among AR and asthmatic patients (p=0.004).

**Table 2:** Short-form allergic rhinitis (SFAR) and ACT analysis SPT: Skin prick test; ACT: Asthma control test.

Analyzed variables	Total population n (%)	Not controlled at all/poorly controlled	Somewhat controlled	Well-controlled	Completely controlled	P-value
Nasal symptoms (apart from a cold or flu) in the past 12 months						0.006
Watery runny nose	69 (24.5%)	9 (3.2%)	22 (7.8%)	27 (9.6%)	11 (3.9%)	
Sneezing (especially violent and in bouts)	52 (18.4%)	10 (3.5%)	13 (4.6%)	23 (8.2%)	6 (2.1%)	
Nasal obstruction	67 (26.6%)	15 (5.3%)	12 (4.2%)	30 (10.6%)	10 (3.5%)	
No, I haven’t	75 (30.6%)	19 (8.7%)	6 (2.4%)	22 (8.3%)	28 (10.7%)	
Where these nasal symptoms accompanied by itchy-watery eyes?						0.024
Yes	165 (52.4%)	33 (9.2%)	42 (12.6%)	65 (21.8%)	25 (8.7%)	
No	117 (41.5%)	20 (7.1%)	17 (6.0%)	47 (16.7%)	33 (11.7%)	
Which season did these nasal symptoms occur (choose all that apply)?						0.264
I do not have nose problems related to certain seasons or months	119 (42.2%)	25 (8.9%)	21 (7.4%)	45 (16.0%)	26 (9.2%)	
Winter season (January, February, December)	68 (24.1%)	9 (2.4%)	17 (6.0%)	29 (10.3%)	13 (4.6%)	
Spring season (March, April)	16 (5.7%)	1 (0.3%)	3 (1.1%)	5 (1.7%)	7 (2.5%)	
Summer season (May, June, July, August, September)	42 (14.9%)	7 (2.5%)	10 (3.5%)	17 (6.0%)	8 (2.8%)	
Autumn/Fall season (October, November)	25 (8.9%)	9 (2.4%)	6 (2.1%)	9 (2.4%)	1 (0.3%)	
Triggering factors (choose all that apply)						0.085
No factors provoked or increased my nose symptoms	36 (12.8%)	9 (3.2%)	2 (0.7%)	14 (6.8%)	11 (5.0%)	
Pollen (from trees, flowers, and grasses)	106 (37.6%)	17 (6.0%)	24 (8.5%)	45 (16.0%)	20 (7.1%)	
House dust mites	84 (29.7%)	14 (5.0%)	15 (5.3%)	31 (11%)	21 (7.4%)	
Dust	18 (6.4%)	4 (1.4%)	6 (2.1%)	7 (2.5%)	1 (0.3%)	
Animals (especially cats and dogs)	11 (3.9%)	2 (0.7%)	1 (0.3%)	6 (2.1%)	2 (0.7%)	
Fungi and mold (inside or outside the house), other (Blank)	17 (6.0%)	4 (1.4%)	6 (2.1%)	7 (2.5%)	1 (0.3%)	
Known to have allergies						<0.001
Yes	228 (75.7%)	53 (80.9%)	59 (20.9%)	112 (39.7%)	58 (20.6%)	
No	54 (19.1%)	8 (2.8%)	3 (1.1%)	18 (6.4%)	25 (8.9%)	
Tested for allergy (SPT, IgE)						0.080
Yes, it was positive	49 (17.4%)	13 (4.6%)	15 (5.3%)	13 (4.6%)	8 (2.8%)	
Yes, it was negative	223 (79.1%)	40 (14.2%)	44 (15.6%)	99 (35.1%)	50 (17.7%)	
No, I have not been tested before	0 (0.0%)	0 (0.0%)	0 (0.0%)	0 (0.0%)	0 (0.0%)	
Doctor already diagnosed that you suffer/suffered from eczema						0.148
Yes	57 (20.2%)	13 (4.6%)	14 (5%)	24 (8.5%)	6 (2.1%)	
No	225 (79.8%)	40 (14.2%)	45 (16.0%)	88 (31.2%)	52 (18.4%)	
Member of your family suffering from asthma, eczema, or allergic rhinitis. If Yes, indicate the person and disease (Tick all that apply).						0.004
No one in my family has these diseases	125 (44.3%)	22 (7.8%)	21 (7.4%)	44 (15.6%)	38 (13.5%)	
Yes, Father (asthma, eczema, or allergic rhinitis)	54 (19.1%)	11 (3.9%)	15 (5.3%)	19 (6.7%)	9 (3.2%)	
Yes, Mother (asthma, eczema, or allergic rhinitis)	42 (14.9%)	4 (1.4%)	13 (4.6%)	23 (8.2%)	2 (0.7%)	
Yes, Siblings (asthma, eczema, or allergic rhinitis)	61 (21.6%)	16 (5.7%)	10 (3.5%)	26 (9.2%)	9 (9.2%)	

Association between allergic rhinitis severity and asthmatic control

The results of the statistical analysis show a significant association between AR severity and asthmatic control. The p-value for the ARIA results is 0.001, indicating the presence of a relationship between these two variables, which implies that symptoms do disturb the majority of the respondents’ activities, sleep, work in school, and daily activities (sports, leisure, etc.) and was troublesome. When looking at the specific categories of AR severity, there are also significant associations with asthmatic control.

Skin Prick Test (SPT)

However, there were no patients with partially controlled asthmatic control in this group. This relationship was also statistically significant with a p-value of 0.001. The results at each level are shown in Table 3.

**Table 3:** Allergic rhinitis severity and asthmatic control ARIA: Allergic Rhinitis and Its Impact on Asthma.

Analyzed variables	Total population n (%)	Not controlled at all	Partially controlled	Controlled	P-value
	n = 49	n = 13	n = 15	n = 21	
ARIA results					0.001
None	6 (12.2%)	2 (15.4%)	1 (6.7%)	3 (14.3%)	0.002
Intermittent mild	9 (18.4%)	4 (30.8%)	1 (6.7%)	4 (19.0%)	0.012
Intermittent (moderate-severe)	17 (34.7%)	3 (23.1%)	7 (46.7%)	7 (33.3%)	0.138
Mild persistent (moderate-severe)	13 (26.5%)	2 (15.4%)	6 (40%)	5 (23.8%)	0.017
Persistent (moderate-severe)	4 (8.2%)	2 (15.4%)	0 (0.0%)	2(9.5%)	0.001

ACT analysis

To assess the relationship, we used a chi-square test of independence. The results indicated that there is no significant association between the effect of the work done at work, school, or at home in the past four weeks; frequency of shortness of breath in the past four weeks; and asthma control based on the data (p > 0.05). However, there was a statistically significant association between the frequency at which asthma symptoms (wheezing, coughing, and shortness of breath) wake you up at night or earlier than usual in the morning (in the past four weeks), frequency of using rescue inhaler or nebulizer medication in the past four weeks, and asthma control (p < 0.05). The results are shown in Table 4.

**Table 4:** Asthma control test (ACT) analysis

Analyzed variables	Total population, n (%)	Not controlled at all or poorly controlled	Somewhat controlled	Well controlled	Completely controlled	P-value
*Effect of the much work done at work, school, or home in the past four weeks*						0.261
All of the time	3 (6.1%)	3 (6.1%)	0 (0.0%)	0 (0.0%)	0 (0.0%)	
Most of the time	3 (6.1%)	5 (10.2%)	6 (12.2%)	3 (6.1%)	1 (2.0%)	
Some of the time	14 (28.6%)	2 (4.8%)	6 (12.2%)	4 (8.2%)	2 (4.8%)	
A little of the time	7 (15.4%)	1 (2.0%)	2 (4.8%)	2 (4.8%)	2 (4.8%)	
None of the time	10 (20.4%)	2 (4.8%)	1 (2.0%)	4 (8.2%)	3 (6.1%)	
*Frequency of shortness of breath in the past four weeks*						0.428
More than once a day	8 (16.3%)	3 (6.1%)	3 (6.1%)	1 (2.0%)	1 (2.0%)	
Once a day	8 (16.3%)	2 (4.8%)	3 (6.1%)	1 (2.0%)	2 (4.8%)	
3 to 6 times a week	8 (16.3%)	3 (6.1%)	3 (6.1%)	2 (4.8%)	0 (0.0%)	
Once or twice a week	16 (32.7%)	4 (8.2%)	4 (8.2%)	7 (15.4%)	1 (2.0%)	
Not at all	9 (18.4%)	1 (2.0%)	2 (4.8%)	2 (4.8%)	4 (8.2%)	
*Frequency at which asthma symptoms (wheezing, coughing, and shortness of breath) wake you up at night or earlier than usual in the morning in the past four weeks*						0.038
4 or more nights a week	8 (16.3%)	5 (10.2%)	1 (2.0%)	1 (2.0%)	1 (2.0%)	
2 to 3 nights a week	5 (10.2%)	1 (2.0%)	3 (6.1%)	0 (0.0%)	1 (2.0%)	
Once a week	7 (15.4%)	1 (2.0%)	3 (6.1%)	2 (4.8%)	1 (2.0%)	
Once or twice a month	12 (24.5%)	4 (8.2%)	4 (8.2%)	4 (8.2%)	0 (0.0%)	
Not at all	17 (34.7%)	2 (4.8%)	4 (8.2%)	6 (12.2%)	5 (10.2%)	
*Frequency of using your rescue inhaler or nebulizer medication (such as albuterol or ventolin) in the past four weeks*						0.001
3 or more times per day	7 (11.5%)	5 (10.2%)	1 (2.0%)	1 (2.0%)	0 (0.0%)	
1 or 2 times per day	8 (16.3%)	2 (4.8%)	3 (6.1%)	3 (6.1%)	0 (0.0%)	
2 or 3 times per week	11 (22.4%)	4 (8.2%)	7 (15.4%)	0 (0.0%)	0 (0.0%)	
Once a week or less	9 (18.4%)	0 (0.0%)	3 (6.1%)	4 (8.2%)	2 (4.8%)	
Not at all	14 (28.6%)	2 (4.8%)	1 (2.0%)	5 (10.2%)	6 (12.2%)	

## Discussion

Respondents’ characteristics

The findings of the study indicate that a significant proportion of asthmatic patients in the Qassim region also suffer from rhinitis. Approximately one-quarter of the participants had been diagnosed with AR by a physician. This suggests a high prevalence of rhinitis comorbidity in asthmatics in the region. The study also found that the majority of participants were female, which may have implications for the relationship between gender and asthma-rhinitis comorbidity. The majority of the respondents (51.06%) were shown to have normal weight, which implies that asthma does not have much effect on both health. Similarly, the majority of them were between 14 and 29 years old, which means the incidences of asthmatic condition is gradually increasing among the Qassim region population based on the fact that the young generation is more affected. Additionally, the majority of participants (65.6%) did not have any chronic pulmonary disease. The study also found that the majority of participants had never smoked at all; however, we did not take into account the effect of those who smoked for a year and above as it was not the major concern of the study. In terms of education, the majority of participants had a college education or above. This may indicate a higher level of awareness and healthcare-seeking behavior among individuals with higher education levels, which could contribute to the higher prevalence of diagnosed rhinitis in this group. In terms of treatment, intranasal corticosteroids were the most commonly used medication for AR, followed by antihistamines and nasal decongestants. The majority of participants reported not taking any medication for their AR, suggesting a potential treatment gap in this population which might have been contributed by financial constraints, ignorance, or the use of traditional methods of treatment. Based on the study by Mahfouz et al., it is noted that patients with AR had a significantly higher risk of experiencing asthma exacerbations compared to those without AR. Over 80% of patients with AR reported experiencing at least one asthma exacerbation in the past year, compared to 72% of patients without AR [7]. The study conducted by Lababidi et al. indicated that the Arabic ACT showed a good correlation with the clinical assessment of asthma control based on Global Initiative for Asthma (GINA) guidelines. The sensitivity of the Arabic ACT was 88.84%, and the specificity was 82.5% for detecting poorly controlled asthma [13].

SFAR and asthmatic control

The findings of the study indicate that there is a significant association between the common AR symptoms and asthma control in the Qassim region. Symptoms such as a runny nose, sneezing, and nasal obstruction are significantly associated with AR and asthmatic symptoms. However, there is no significant association between nose problems occurring in specific seasons or months and AR and asthmatic symptoms, suggesting that seasonality may not have a strong impact on asthma control. House dust mite allergies have a borderline significant association with AR and asthmatic symptoms. Having a family history of asthma, eczema, or AR is associated with AR and asthmatic symptoms, thus indicating a significant impact on asthma control. Overall, these findings suggest that AR plays a role in asthma control, with certain symptoms and allergies having a stronger association. Understanding and addressing these comorbidities may be essential to effectively manage asthma in individuals in the Qassim region. This concurs with the findings obtained by de Andrade et al. in their study, which noted that there is a high prevalence of comorbidity between asthma and AR among adolescents. The findings of the study reveal that approximately 70% of the participants with asthma also had AR, and 57% of participants with AR also had asthma. This suggests that there is a strong association and potential shared underlying mechanisms between these two conditions in this age group [14]. Ohta et al. in their study also found that the prevalence of rhinitis in asthmatic patients in Japan is high, with 84.5% of asthmatics also reporting rhinitis symptoms. The study further reported that rhinitis symptoms have a significant impact on the quality of life of asthmatic patients, with higher rates of sleep disturbance, fatigue, and impairment in daily activities [15].

ARIA and asthmatic control

The results of the statistical analysis show a significant association between AR severity and asthmatic control. The study noted that symptoms do disturb the majority of the respondents’ activities, including sleep, work in school, and daily activities (sports, leisure, etc.) and were troublesome. In summary, the results indicate that as the severity of AR increases, the level of asthmatic control tends to decrease. This suggests a possible link between the two conditions and emphasizes the importance of managing both conditions in order to achieve optimal control. This corresponds with Stern et al.'s study, which noted that there is a significant association between AR and asthma outcomes in city schoolchildren. The study found that children with both AR and asthma had poorer asthma control, increased asthma symptoms, and higher healthcare utilization compared to children with asthma alone. This suggests that managing AR could potentially improve asthma outcomes in this population [16].

Further, the findings of the study suggest that there is no significant association between the amount of work done at work, school, or home in the past four weeks, the frequency of shortness of breath in the past four weeks, and asthma control based on the data. On the other hand, the study revealed that there is a statistically significant association between the frequency at which asthma symptoms (wheezing, coughing, and shortness of breath) wake you up at night or earlier than usual in the morning in the past four weeks, the frequency of using your rescue inhaler or nebulizer medication in the past four weeks, and asthma control, indicating that the observed associations between these two factors and asthma control were statistically significant and not due to chance. Ciprandi et al.'s main finding of their study is that patient-related factors, such as duration of disease and the presence of comorbidities, can significantly impact the satisfaction of patients with allergy treatment for rhinitis and asthma. The study found that younger patients, female patients, patients with shorter disease durations, and patients without comorbidities were more satisfied with their allergy treatment. These findings highlight the importance of considering patient characteristics and preferences when developing and delivering allergy treatment strategies [17].

Limitations of the study

The study was limited to evaluating the prevalence and severity of rhinitis comorbidity in asthmatics patients utilizing asthma medication in the Qassim region and identifying whether rhinitis affects control; hence, it did not take into account the effect of smoking for more than one year and ex-smokers who have stopped in the past one year. Additionally, the study concentrated on the general effect of allergy rhinitis on asthma control without considering the effect across the participants of different ages. Further, the presence of a high percentage of the participants with rhinitis fully controlled could give biased results. More so, statistical tests used can only provide significance without commenting on the strength of the association.

## Conclusions

In summary, this study found a high prevalence of rhinitis symptoms comorbidity in asthmatic patients in the Qassim region. Overall, the study established the existence of a relationship between AR and asthmatic control. Symptoms such as a runny nose, sneezing, and nasal obstruction are significantly associated with AR and asthmatic symptoms. However, there is no significant association between nose problems occurring in specific seasons or months and AR and asthmatic symptoms, suggesting that seasonality may not have a strong impact on asthma control. House dust mite allergies have a borderline significant association with AR and asthmatic symptoms. Having a family history of asthma, eczema, or AR is associated with AR and asthmatic symptoms, thus indicating a significant impact on asthma control.
